# Glycosylation-mediated phenylpropanoid partitioning in *Populus tremuloides *cell cultures

**DOI:** 10.1186/1471-2229-9-151

**Published:** 2009-12-29

**Authors:** Raja S Payyavula, Benjamin A Babst, Matthew P Nelsen, Scott A Harding, Chung-Jui Tsai

**Affiliations:** 1 School of Forest Resources and Environmental Science, Michigan Technological University, Houghton, MI 49931, USA; 2 Warnell School of Forestry and Natural Resources, University of Georgia, Athens, GA, 30602, USA; 3 Department of Genetics, University of Georgia, Athens, GA, 30602, USA; 4 Current address: United States Department of Agriculture, Agricultural Research Services, Prosser, WA 99350, USA; 5 Current address: Committee on Evolutionary Biology, University of Chicago, Chicago, IL 60637, USA

## Abstract

**Background:**

Phenylpropanoid-derived phenolic glycosides (PGs) and condensed tannins (CTs) comprise large, multi-purpose non-structural carbon sinks in *Populus*. A negative correlation between PG and CT concentrations has been observed in several studies. However, the molecular mechanism underlying the relationship is not known.

**Results:**

*Populus *cell cultures produce CTs but not PGs under normal conditions. Feeding salicyl alcohol resulted in accumulation of salicins, the simplest PG, in the cells, but not higher-order PGs. Salicin accrual reflected the stimulation of a glycosylation response which altered a number of metabolic activities. We utilized this suspension cell feeding system as a model for analyzing the possible role of glycosylation in regulating the metabolic competition between PG formation, CT synthesis and growth. Cells accumulated salicins in a dose-dependent manner following salicyl alcohol feeding. Higher feeding levels led to a decrease in cellular CT concentrations (at 5 or 10 mM), and a negative effect on cell growth (at 10 mM). The competition between salicin and CT formation was reciprocal, and depended on the metabolic status of the cells. We analyzed gene expression changes between controls and cells fed with 5 mM salicyl alcohol for 48 hr, a time point when salicin accumulation was near maximum and CT synthesis was reduced, with no effect on growth. Several stress-responsive genes were up-regulated, suggestive of a general stress response in the fed cells. Salicyl alcohol feeding also induced expression of genes associated with sucrose catabolism, glycolysis and the Krebs cycle. Transcript levels of phenylalanine ammonia lyase and most of the flavonoid pathway genes were reduced, consistent with down-regulated CT synthesis.

**Conclusions:**

Exogenous salicyl alcohol was readily glycosylated in *Populus *cell cultures, a process that altered sugar utilization and phenolic partitioning in the cells. Using this system, we identified candidate genes for glycosyltransferases that may mediate the glycosylation, and for transporters that mediate the subcellular compartmentalization of sugars and phenolic glycosides. The suspension cells appear to represent a facile system for dissecting the regulation of phenolic carbon partitioning, and in turn, its effects on growth in *Populus*.

## Background

*Populus *trees and their close relatives in the family *Salicaceae *are capable of producing large and varying quantities of phenolic glycosides (PGs) and condensed tannins (CTs) in their vegetative tissues [1,2]. PGs and CTs play a major role in defense and protection against biotic (e.g. herbivore) [3,4] and abiotic (e.g. UV-B radiation) [5] stress. Both PGs and CTs are thought to be derived from the phenylpropanoid pathways [6]. Salicin (salicyl alcohol glucoside, *O*-hydroxymethyl phenyl β-D-glucoside), the first PG-like substance isolated from *Salix *bark, is considered a potential precursor of higher-order PGs, such as salicortin and tremulacin [2,7,8]. CTs are polyphenolics composed of proanthocyanidin monomers, and are derived from the flavonoid pathway initiated by the conjugation and modification of phenylpropanoid and malonyl-CoA [6,9,10].

Several cases suggest a possible negative correlation between CT and PG biosynthesis. For example, there is an ontogenetic shift from high PG and low CT levels in leaves of young *Populus *plants, to low PG and high CT levels as trees age [11]. In both *Populus *and *Salix*, there are examples of species that accumulate high CTs, with little or no PGs (e.g. *P. angustifolia, S. eriocephala*), while others accumulate high PGs and low CTs (e.g., *P. fremontii *and *S. sericea*) [12,13]. Negative correlations of PGs and CTs have also been observed in inter-specific hybrids within these genera (e.g., *P. fremontii *× *angustifolia *and *S. sericea *× *eriocephala*) [12-14].

Accumulation of high levels of PGs and CTs has a negative effect on growth, especially under low nutrient conditions [4]. A tradeoff between growth and the synthesis of salicylates was also observed in *in vitro *micropropagated *Salix *plantlets [2]. This is not surprising, given that, in *Populus *for example, the total levels of PGs and CTs may reach 30% dry wt. The synthesis of growth-compromising levels of these compounds may be necessary for defense, although it is an added cost to the plant. The molecular mechanisms underlying the metabolic competition between growth and nonstructural phenylpropanoids, and between the different phenylpropanoid pools are not known.

Since the genes involved in PG biosynthesis have not yet been identified, a transgenic approach is not currently feasible. Stable-isotope feeding experiments suggest that biosynthesis of salicin depends on 2-hydroxylated phenolic precursors (Babst, Harding and Tsai, submitted). We therefore developed a *Populus *cell suspension culture to use in feeding experiments with several 2-hydroxylated phenolic derivatives to manipulate PG synthesis. We determined that *Populus tremuloides *cell cultures produce CTs but not PGs under normal culture conditions, and that cells are capable of producing salicin by glycosylating exogenous salicyl alcohol or salicylaldehyde. A series of experiments exploiting the glycosylation capacity of the cells revealed a metabolic tradeoff between salicin accumulation and CT formation. Microarrays were used to determine changes in gene expression across different categories of metabolic pathways, and to gain insights about the consequences of salicyl alcohol glycosylation to secondary carbon partitioning. Investigation of metabolic shifts associated with PG homeostasis in simple cell culture systems is expected to yield clues about regulation of PGs at the whole organ or plant levels.

## Results

### Growth and secondary metabolite production of the aspen cell culture

**Figure 1:**
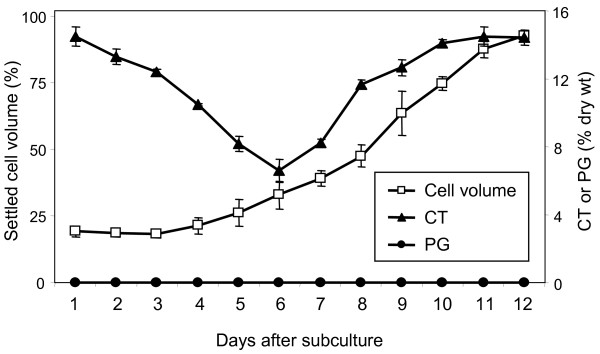
**Growth and secondary metabolite level in *Populus *L4 cell suspensions grown under normal culture conditions**. Cell growth was estimated as the percent settled cell volume (the fraction of the suspension occupied by the cells, shown on the left axis) using nephelo flasks. CT and PG concentrations (percent dry weight, shown on the right axis) were determined by the Porter assay and HPLC, respectively. Data represents the mean ± SE of three biological replicates.

Woody species, including *Populus*, are generally recalcitrant to cell suspension culture. We have previously identified an aspen (*Populus tremuloides *Michx.) clone (L4) that is amenable to suspension culture from a screen of over 200 seeds (Tsai, unpublished data). This "suspension culture" trait appears to be genetically rather than developmentally controlled, as friable calli can also be routinely induced from young leaves of vegetatively propagated L4 plants maintained in a greenhouse. A typical growth curve of the L4 cell line is shown in Fig 1. With an 11-day subculture interval, the cell volume usually increased by ~400% at the end of the culture cycle. This pattern of cell growth was stable for more than 40 cycles during the experimental period.

The cells were harvested at regular intervals to determine the basal CT and PG concentrations during the culture cycle. At the start of each culture period (i.e., after subculture), CT concentrations averaged ~14.5% dry wt, but decreased gradually through the lag and early log phases to a low of ~6.5%, and then increased rapidly thereafter, reaching ~15% at the end of the culture cycle (Fig. 1). The initially high CT content corresponded with the CT level at the end of the culture cycle. The gradual decrease in CT content during early growth may be suggestive of CT breakdown and a lack of or little CT synthesis. Under normal culture conditions, the L4 cell line did not accumulate salicin or any other higher-order PGs.

### Feeding of potential salicin precursors

**Figure 2:**
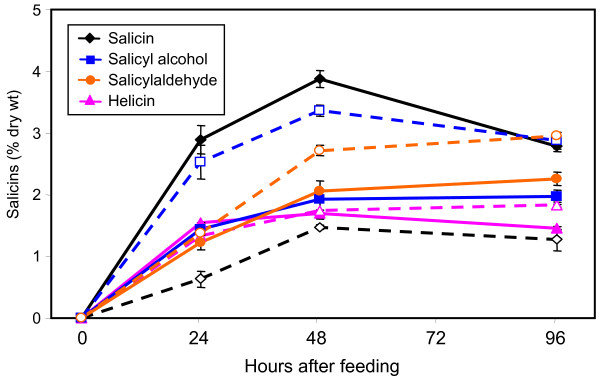
**Levels of salicin and isosalicin accumulation in cultures fed with 1 mM precursors**. Solid and dotted lines represent salicin and isosalicin levels, respectively, detected over a 96 h period in cultures fed with salicin, helicin, salicyl alcohol and salicylaldehyde. Error bars represent the measurement range of two biological replicates.

Because PGs (salicin, salicortin and tremulacin) are detected in the leaves of the aspen L4 line, the inability of cultured cells to produce PGs may be attributed to the absence of PG precursors and/or spatiotemporal regulation of metabolic activities in suspension cells versus intact tissues. Feeding experiments were undertaken to test whether PGs would accumulate if putative phenolic precursors were supplied. Phenolic acids (benzoic acid, cinnamic acid, *O-*coumaric acid and salicylic acid) were fed at 0.2 mM, since higher feeding levels led to cell browning in preliminary trials, suggestive of toxicity. Phenolic alcohols (salicyl alcohol and benzyl alcohol), aldehydes (benzylaldehyde and salicylaldehyde) and glucosides (salicin and helicin) were fed at 1 mM. Feeding was conducted 5 days after subculture (DAS), at early log phase, and cells were harvested after 24, 48 and 96 h. The glucoside salicin was readily taken up and detected primarily as unaltered salicin in the cell extracts, although a slow conversion to its unnatural isomer, isosalicin, continued throughout the 96 hr feeding period (Fig. 2, Additional file 1). Feeding salicyl alcohol (the aglycone of salicin), salicylaldehyde and helicin (salicylaldehyde glucoside) also resulted in the accumulation of both salicin and isosalicin (hereafter referred to as salicins). In all cases, accumulation of salicins reached a plateau by 48 hr (Fig. 2), and was higher with salicin and salicyl alcohol feeding, than with salicylaldehyde and helicin feeding. Higher-order PGs were not detected during any of the feeding experiments. Feeding with all other compounds led to accumulation of their respective glucosides (see Additional file 1), but not PGs, and these compounds were not studied further. The feeding results showed that the cells are capable of accumulating both CTs (under normal condition) and simple PGs (upon precursor feeding), suggesting that the cell culture system can be exploited for the investigation of metabolic competition. Salicyl alcohol was chosen for subsequent feeding experiments because it led to a higher accumulation of salicins

### Dose-dependent effects of salicyl alcohol feeding

**Figure 3:**
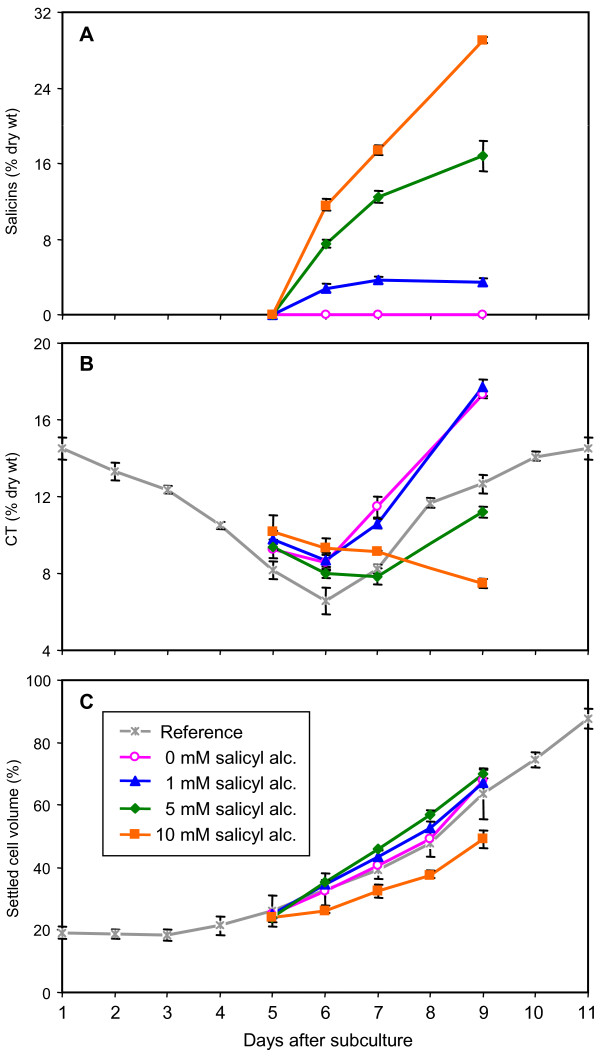
**Dose-dependent effects of salicyl alcohol feeding on secondary metabolite accumulation and growth in cell cultures**. Salicyl alcohol at 0, 1, 5, or 10 mM was fed to 5-day old cultures and samples were analyzed for (A) total salicins, (B) CTs and (C) growth over a 96 h period. The grey lines in panels B and C are reference data points from separate control samples as presented in Figure 1. Error bars represent the measurement range of two biological replicates.

When cells were fed varying levels of salicyl alcohol (0, 1, 5 and 10 mM), a dose-dependent accumulation of salicins was observed (Fig. 3A). Accumulation of salicins plateaued 24 h and 48 h after feeding with 1 mM and 5 mM salicyl alcohol, respectively, but continued to increase with 10 mM feeding over the 4-day monitoring period. The total salicins detected in the cells at the end of the experiment were 3, 17 and 29% dry weight with 1, 5, and 10 mM salicyl alcohol, respectively. At 5 mM feeding, the efficiency of salicyl alcohol uptake and conversion at the end of the 4-day period was estimated to be ~60%, as 175 μmoles of salicyl alcohol were converted to 100 μmoles of salicins.

CT levels showed a negative dose-dependent response to salicyl alcohol feeding. With 1 mM feeding, CT levels were similar to those of unfed cultures, in which CT levels decreased after 24 h (corresponding to 6 DAS), followed by a steady increase through the end of the 96 h period (Fig. 3B). This is consistent with the CT accrual kinetics typical of control cells (Fig. 1, also shown as the gray line in Fig. 3B), where CT abundance declines to a minimum at 6 DAS before exhibiting a recovery. Feeding at 5 mM caused the CT levels to decrease through 48 h (or 7 DAS), and delayed the normal pace of CT accrual by ~2 days. At 10 mM, cells showed a downward trend of CT levels and did not resume CT synthesis throughout the 96 h feeding period. At this feeding level, cell growth was also reduced by up to 28% (Fig. 3C). Together, these results are consistent with a negative effect of salicyl alcohol glycosylation on the accumulation of CTs.

### Effects of culture age on salicyl alcohol feeding responses

**Figure 4:**
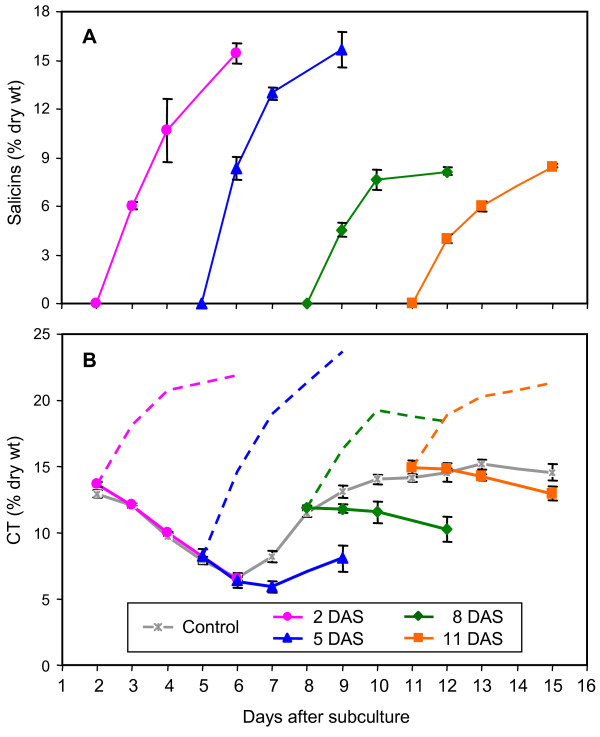
**Effects of culture age on secondary metabolite accumulation following 5 mM salicyl alcohol feeding**. Salicyl alcohol at 5 mM was fed to cells 2, 5, 8, or 11 days after subculture (DAS) and samples were analyzed for (A) total salicins, and (B) CTs over a 96 h period. In panel B, the grey line represents CT levels from control (unfed) cultures of this experiment. Dashed color lines represent total phenolics (salicins plus CTs) levels for the corresponding samples. Error bars represent the measurement range of two biological replicates.

To examine whether culture growth phase plays a role in cellular capacity to accumulate secondary metabolites, cultures at 2, 5, 8, or 11 DAS were fed 5 mM salicyl alcohol and sampled over a 96 h period. In general, cells at the lag (2 DAS) or early exponential (5 DAS) phases, when the CT levels were in decline, exhibited a higher capacity to accumulate salicins than cells at the mid-exponential (8 DAS) or stationary (11 DAS) phases when CT levels were increasing (Fig. 4A). At the end of the experimental period, total salicins reached ~15% and ~8% in cells fed at the earlier and later stages, respectively.

Salicyl alcohol feeding at the lag phase (2 DAS) had no effect on basal CT concentration (Fig. 4B). Feeding at 5 DAS delayed the onset of CT accumulation as described above (Fig. 3B), and reduced CT levels by 38% relative to unfed controls (Fig. 4B). At later stages of cell growth (8 and 11 DAS) when CT levels were high, salicyl alcohol feeding abolished further CT increase. These results provide support for a reciprocal metabolic competition between CT synthesis and salicyl alcohol glycosylation at several stages of culture growth.

### Microarray analysis

The observed reciprocal regulation of salicins and CTs in cell cultures suggested that salicyl alcohol feeding may have affected both carbohydrate utilization and carbon flux through the phenylpropanoid pathway. To gain additional information about the metabolic response, gene expression analysis was carried out using a custom aspen 7K EST array. Cells were fed either 5 mM salicyl alcohol or water at 5 DAS, and harvested after 48 h for the analysis. The 48 h time point (corresponding to 7 DAS) was chosen because CT levels typically begin to increase by this time in unfed cells from a minimum at 6 DAS (Figs. 1 and 4B); CT levels did not exhibit the normal increase and remained near the minimum in the fed cells at 7 DAS (Fig. 4B); and accumulation of salicins in the fed cells reached its plateau after 48 h (Figs. 2, 3 &4). The 48 h feeding window therefore provided an ideal metabolic context with a reproducible difference between salicyl alcohol-fed and control cells for assessing the underlying transcriptome adjustments.

Of the 3,957 ESTs that passed a series of quality control measures, expression of 725 ESTs representing 659 non-redundant genes was found to be significantly altered (false discovery rate *p *≤ 0.05) by salicyl alcohol feeding (see Methods). Most of these genes showed small expression changes, and only 35 ESTs exhibited ≥ 2-fold differences. This limited transcriptome change was not unexpected, given the observation that glycosylation of exogenous salicyl alcohol led to a delay in CT increase with no apparent effect on cell growth under the condition (48 h post feeding) studied. For further analysis, we applied a modest fold-change cutoff of 1.3 yielding 284 and 160 ESTs that were up- and down-regulated, respectively (Additional file 2).

Stress-related transcripts, such as glutathione S transferase, dehydrin, thaumatin-like protein, and germin-like protein, were among the most up-regulated genes in the salicyl alcohol-fed cells (Table 1). The most highly up-regulated EST (MTU6CR.P6.H02) corresponded to a peroxidase. This gene is poorly expressed in vegetative tissues, except cell cultures, and is strongly up-regulated in methyl jasmonate-treated cells as well as in insect-damaged aspen leaves [15] (Tsai, unpublished data). Many of the down-regulated genes were associated with phenylpropanoid and flavonoid biosynthesis (Table 1), including phenylalanine ammonia-lyase, 4-coumurate:CoA ligase, caffeoyl-CoA *O*-methyltransferase, cinnamoyl-CoA reductase, and leucoanthocyanidin reductase. These results are in line with the reduced CT accumulation in salicyl alcohol-treated cells.

**Table 1:** List of representative ESTs differentially regulated in salicyl alcohol-fed cultures.

EST ID	Putative function	JGI Gene Model	Ratio	Adjusted *P*-value
***Defense***				
MTU6CR.P6.H02	Peroxidase	estExt_fgenesh4_pm.C_870009	8.18	< 0.001
MTU6TR.P1.H11	Glutathione S-transferase	grail3.0036009801	4.37	< 0.001
MTU6TR.P12.E08	Dehydrin	estExt_fgenesh4_pg.C_LG_V1612	3.13	< 0.001
MTU6CR.P6.E04	Osmotin-like protein	gw1.I.8918.1	2.77	< 0.001
MTU6CR.P18.H10	Pollen Ole e 1 allergen and extensin family protein	eugene3.00040501	2.62	0.006
MTU6CR.P17.A06	Germin-like protein	estExt_fgenesh4_pm.C_LG_XIII0003	2.09	0.015
MTU6TR.P2.F05	hypersensitive-induced reaction protein	estExt_fgenesh4_pg.C_LG_XVII0326	2.07	0.006
MTU6CR.P15.D05	Germin-like protein	gw1.131.45.1	1.99	0.001
MTU6TR.P1.D06	Polyphenol oxidase	eugene3.00110271	1.92	0.001
MTU6CR.P11.H04	Cysteine protease inhibitor	gw1.IX.4482.1	1.88	0.002
MTU7CL.P2.B11	Carbohydrate oxidase, antifungal	gw1.I.5965.1	1.81	< 0.001
MTU5CS.P13.E03	Harpin-induced protein	gw1.VI.811.1	1.71	< 0.001
MTU4CA.P21.G12	Thaumatin-like protein	gw1.I.9073.1	1.62	0.001
MTU6CR.P14.C11	Glutathione reductase	estExt_Genewise1_v1.C_LG_III0933	1.58	0.002
MTU7TL.P6.C04	Disease resistance protein	estExt_fgenesh4_pg.C_LG_II1171	1.55	0.001
MTU3TS.P13.B07	Metallothionein	eugene3.00091335	1.50	0.020
MTU6CR.P16.G06	Chitinase-like protein	estExt_fgenesh4_pm.C_LG_XIV0394	1.48	0.001
MTU2CA.P11.E09	CuZn-superoxide dismutase	eugene3.00700152	1.47	0.028
MTU2TA.P7.F06	CuZn-superoxide dismutase	estExt_Genewise1_v1.C_LG_XIII1233	1.36	0.046
MTU5CS.P17.E01	Hypersensitive-induced response protein	eugene3.00170326	0.75	0.037
MTU4TA.P24.C04	disease resistance protein	estExt_fgenesh4_pg.C_400229	0.72	0.037
MTU7CL.P4.C10	Latex-like protein	estExt_Genewise1_v1.C_LG_X6115	0.64	0.028
				
***Glycolysis and TCA cycle***				
MTU4TA.P27.C06	Aconitase	estExt_fgenesh4_pg.C_LG_II2062	1.74	0.005
MTU6CR.P8.F06	Enolase	eugene3.00151093	1.59	0.031
MTU5CS.P4.B07	Glyceraldehyde 3-phosphate dehydrogenase	estExt_fgenesh4_pg.C_LG_X0484	1.52	0.023
MTU4CA.P22.C01	Transaldolase	grail3.0047006401	1.44	0.015
MTU7TL.P15.C08	Phosphoglycerate kinase	estExt_fgenesh4_pm.C_LG_VIII0335	1.36	0.025
MTU6TR.P1.A05	Malate dehydrogenase	estExt_Genewise1_v1.C_LG_I4975	1.35	0.039
MTU2TA.P7.B04	Phosphoglycerate mutase	estExt_fgenesh4_pg.C_LG_XVI1334	1.34	0.034
MTU2CA.P15.F06	Isocitrate dehydrogenase	grail3.0038019202	1.30	0.023
				
***Sugar metabolism***				
MTUNUL1.P10.C04	Fructose-1,6-bisphosphatase	estExt_fgenesh4_pg.C_LG_VIII0539	1.45	0.007
MTU5CS.P7.G01	Vacuolar invertase	estExt_fgenesh4_pg.C_LG_III0902	2.22	0.006
MTU5CS.P10.C09	Sucrose synthase	estExt_fgenesh4_pg.C_280066	1.34	0.015
				
***Phenylpropanoid metabolism***				
MTU6CR.P7.E07	Phenylalanine ammonia-lyase	fgenesh4_pg.C_LG_X002043	0.67	0.042
MTUNUL1.P25.A01	Cinnamoyl-CoA reductase	estExt_fgenesh4_kg.C_LG_III0056	0.60	0.002
MTU5CS.P10.C05	S-adenosylmethionine synthase	grail3.0050014702	0.57	< 0.001
MTU5CS.P3.F03	S-adenosylmethionine synthase	estExt_fgenesh4_pm.C_LG_XIV0257	0.55	0.005
MTUNUL1.P46.F06	Caffeoyl-CoA O-methyltransferase	grail3.0001059501	0.43	0.002
MTU2TA.P2.H05	Phenylalanine ammonia-lyase	estExt_Genewise1_v1.C_280658	0.43	0.010

Genes encoding fructose 1,6-bisphosphatase and sucrose-hydrolyzing enzymes, such as sucrose synthase (SuSy) and vacuolar invertase (VIN), were up-regulated by salicyl alcohol feeding (Table 1). Also up-regulated were genes comprising the glycolysis pathway, including glyceraldehyde 3-phosphate dehydrogenase, phosphoglycerate kinase, phosphoglycerate mutase, and enolase, and genes comprising the tricarboxylic acid (TCA, Krebs) cycle, including malate dehydrogenase, aconitase and isocitrate dehydrogenase (Table 1).

### Real-time PCR analysis

QPCR was used to enable higher resolution analysis of the gene expression changes observed in microarrays. Because of the limited coverage of the EST array, we expanded the analysis to include all known flavonoid biosynthetic pathway genes [6], as well as gene families associated with the transport and hydrolysis of sucrose, and the glycosylation and transport of simple phenolics [16-20].

#### Phenylpropanoid and flavonoid genes

**Figure 5:**
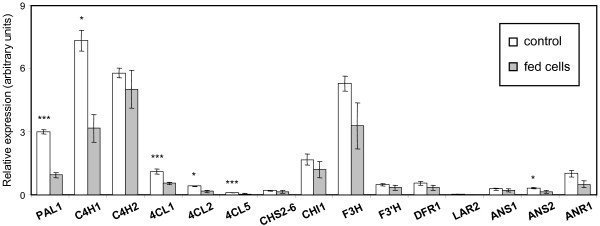
**Q-PCR expression analysis of phenylpropanoid and flavonoid pathway genes**. Relative transcript levels in control and salicyl alcohol-fed cultures are presented as white and gray bars, respectively. Data represents the mean ± SE of three biological replicates. Significance of differences between control and fed cultures was determined using the two-sample *t*-test, as indicated by asterisks (*P < 0.1; **P < 0.05; ***P < 0.01). Expression of the following genes was near or below detection limit, and was not shown: *PAL2*, *PAL3, PAL4/5, 4CL3*, *4CL4*, *CHS1*, *F3'5'H*, *ANR2*, *LAR1*, and *LAR3*.

Among the more highly expressed phenylpropanoid and flavonoid genes, transcript levels of *PAL1*, *C4H1*, *4CL1*, *CHI1*, *F3H *and *ANR1 *clearly decreased, with the first three showing statistically significant differences (Fig. 5). *C4H2 *is a paralog of *C4H1*, arising from genome-wide duplication [21], and appears to exhibit differential regulation in response to salicyl alcohol feeding. Expression of several of the other less abundant genes, such as *4CL2, 4CL5 *and *ANS2*, was also significantly reduced. Although the degree of reduction was small in some cases (20-50% among those that were not statistically significant), the trend towards down-regulation was consistent throughout the pathway, confirming the microarray findings.

#### Sucrose metabolism genes

**Figure 6:**
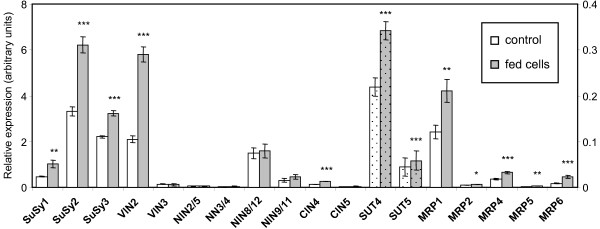
**Q-PCR expression analysis of sucrose synthase, invertase and selected transporter gene family members**. Relative transcript levels in control and salicyl alcohol-fed cultures are presented as white and gray bars, respectively. Data represents the mean ± SE of three biological replicates. Refer to the right-hand side axis for the dotted bars. Significance of differences between control and fed cultures was determined using the two-sample *t*-test, as indicated by asterisks (*P < 0.1; **P < 0.05; ***P < 0.01). Expression of the following genes was near or below detection limit, and was not shown: *SuSy4, SuSy5, SuSy6, VIN1, CIN1/2, CIN3, SUT1/2, SUT3 *and *MRP3*.

Of the six SuSys, five cell wall invertases (CIN), three vacuolar invertases (VIN), and sixteen cytosolic neutral/alkaline invertases (*NIN*) annotated in the poplar genome [21,22], *SuSy2, SuSy3, VIN2 *and *NIN8/12 *were the most strongly expressed in cell cultures (Fig. 6). In support of the microarray results, expression of *SuSy1*, *SuSy2*, *SuSy3 *and *VIN2 *was significantly up-regulated in salicyl alcohol-fed cultures. Transcript levels of all other invertases were either low or did not change (except for *CIN4*) by salicyl alcohol feeding. Of the six sucrose transporter (*SUT*) genes found in the *Populus *genome, only *SUT4 *and, to a much lesser extent, *SUT5 *were detected in cell cultures. Expression of *SUT4 *was significantly up-regulated in salicyl alcohol-fed cells (Fig. 6). *SUT4 *encodes a group III sucrose transporter that exhibits sequence similarity to vacuole-localized *Arabidopsis *AtSUT4 [23] and lotus (*Lotus japonicus*) LjSUT2 [19] (Payyavula et al., in preparation). These results suggested that salicyl alcohol feeding stimulated sucrose hydrolysis in both cytosol and vacuole, as well as sucrose transport across the vacuolar membrane of cultured cells.

#### Glycosyltransferase genes

**Figure 7:**
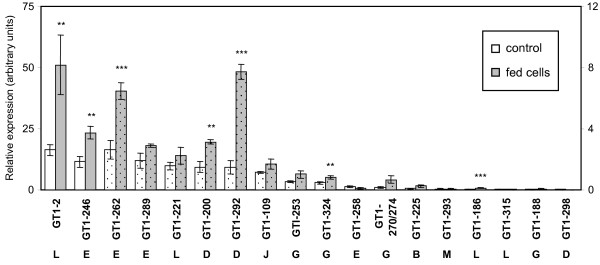
**Q-PCR expression analysis of GT-1 gene family members**. Relative transcript levels in control and salicyl alcohol-fed cultures are presented as white and gray bars, respectively. The arrow at the bottom points to the subgroup of the GTs. Data represents the mean ± SE of three biological replicates. Refer to the right-hand side axis for the dotted bars. Significance of differences between control and fed cultures was determined using the two-sample *t*-test, as indicated by asterisks (*P < 0.1; **P < 0.05; ***P < 0.01). Expression of the following genes was near or below detection limit, and was not shown: *GT1-184, GT1-218, GT1-255*.

The glycosylation of small phenolics is typically catalyzed by members of the glucosyl transferase (GT) family 1 [20]. Of the 326 GT1 family members annotated in the *Populus *genome [24], 47 were expressed in the aspen cell cultures based on Affimetrix microarray analysis [15]. We further narrowed the list to focus on the GT1 sub-families B, D, E and L, as they contain members capable of forming glucose esters (sub-family L) or *O*-glucosides (sub-families B, D and L) of phenolics in *Arabidopsis *[25]. As references, we also included members from sub-families G, J and M that were well-expressed in leaves and co-regulated with foliar phenolics in several *Populus *genotypes subjected to various treatments (e.g., N-stress, wounding; Babst, Harding and Tsai, unpublished). Altogether, a total of 18 *GT1 *genes were selected for QPCR analysis. In general, members of the sub-families D, E and L were expressed at higher levels than those of sub-families B, G, J and M (Fig. 7). In most cases, expression of these *GT1 *members was up-regulated by salicyl alcohol feeding. Transcript levels of *GT1-2 *and *GT1-246 *from sub-families L and E, respectively, were particularly abundant in cell cultures, and were significantly up-regulated 2- to 3-fold by salicyl alcohol feeding. Their strong expression and inducibility suggest that they may be involved in glycosylation of salicyl alcohol.

#### Possible phenolic transporters

Phenolics glycosylated in cytoplasm may be transported into the vacuole. This is thought to be mediated by ATP-binding cassette ABC transporters or H^+^-antiporters, depending on the compound being compartmentalized and the plant species [26-28]. Microarray results showed a slight up-regulation of an EST encoding a Mg-ATP-dependent glutathione conjugate pump, known as MRP transporter, in salicyl alcohol-fed cultures (see Additional file 2). Therefore, we expanded our QPCR analysis to the four putative tonoplast-localized and two plasma membrane-localized MRP transporters identified in the *Populus *genome based on sequence similarity with *Arabidopsis *and maize MRPs [28,29]. *MRP1 *was the most abundant member in cultured cells and was significantly up-regulated by salicyl alcohol (Fig. 6). The expression of *MRP4 *and *MRP6 *was lower in unfed cultures, but was also significantly up-regulated by salicyl alcohol feeding. The other three *MRP *isoforms were poorly expressed in cultured cells. SUTs from several plant species are also capable of transporting a wide range of α- and β-phenolic glucosides, in addition to sucrose, across biological membranes [19,30,31]. Together, these results suggest the possible involvement of *MRP1*, *MRP6 *and *SUT4 *in the transport of salicins into and out of vacuole in aspen cell cultures.

Overall, we noted that the magnitude of the difference revealed by QPCR was 20-50% greater than found by microarray analysis (for a subset of genes where both data types were available, *e.g*., *PAL1*, *C4H1*, *4CL1*, *SuSy2*, *VIN2*, *SUT4*, *GT1-225 *and *MRP1*). The discrepancy was likely due to the underlying differences (fragment hybridization vs. PCR-based detection) of the two techniques. The presence of paralogous genes in many of the gene families, due to genome-wide duplications in *Populus *[6,21], could also have contributed to non-specific signal during EST microarray hybridization.

## Discussion

PGs and CTs are the two most abundant non-structural phenylpropanoid derivatives found in *Populus *plants [4,32]. Here, we showed that CTs are also abundant in aspen cell cultures, but that salicin and higher-order PGs are absent. By supplying putative intermediates, we were able to restore synthesis of salicin, the simplest PG, in the aspen cell cultures, although higher-order PGs remained undetected. All phenolic compounds tested were glycosylated in the cell cultures, but only salicyl alcohol, salicylaldehyde and helicin led to accumulation of salicins. The results are consistent with the interpretation that glycosylation is an active process in cell cultures, and that the capacity for glycosylation rapidly increases in response to phenolic feeding. The cells also have a large capacity to reduce aldehyde groups to their corresponding alcohols, judging from the efficient accumulation of these substrates as salicin. Salicyl alcohol, salicylaldehyde and helicin have all been implicated as immediate or penultimate precursors of salicin in the PG biosynthetic pathways postulated previously [2,33]. The results suggest that neither salicyl alcohol nor salicylaldehyde are produced by heterotrophically cultured aspen cells. Therefore it appears that the lesion preventing PG accumulation may lie upstream of a putative step for catalyzing 2-hydroxylation of benzyl/cinnamyl ring precursors. Due to this likelihood, we decided to exploit the glycosylating capacity of the cells upon precursor feeding, and to focus on the effect of salicyl alcohol glycosylation on potentially competing pathways in growth and secondary metabolism. During the feeding experiments, it was evident that glycosylation of salicyl alcohol interfered with CT biosynthesis, and vice versa. Several experiments were therefore conducted to investigate the relationship between salicyl alcohol feeding, CT accrual and cell growth.

### PG-CT pathway interactions in salicyl alcohol-fed cultures

The reciprocal competition between salicin and CT accumulation was observed under multiple feeding conditions, but varied depending on the physiological/metabolic status of the cultures. Cells exhibited a 2-fold higher capacity to accumulate salicins during early growth phases, when new CT biosynthesis was not apparent, than in mid-late growth phase cells containing high levels of CTs. Conversely, the trajectory of CT accumulation was at most only slightly impacted by salicin accrual at early growth phases, but was clearly compromised when salicyl alcohol was fed at mid-late growth phases. In other words, aspen cells responded to salicyl alcohol feeding by producing PGs and CTs in a compensatory rather than additive fashion, raising the possibility that there was a metabolic constraint to phenylpropanoid biosynthesis. Feeding methyl jasmonate, an elicitor known to stimulate phenylpropanoid gene expression [34], increased CT accumulation, but had no effect on salicyl alcohol glycosylation. Conversely, 5 mM salicyl alcohol feeding reduced the CT accumulations by 63-69% after 48 h, regardless of the methyl jasmonate levels co-administered, at 0, 1 μM or 25 μM (Payyavula, Tsai and Harding, unpublished). These results support a PG-CT tradeoff in salicyl alcohol-fed cells that is independent of the phenylpropanoid biosynthetic capacity.

In the present investigation, the PG-CT tradeoff appears to reveal a sensitivity of phenylpropanoid carbon partitioning to induced glycosylation, since the combined concentrations of CTs plus salicins in the fed cells differed little between culture ages at the end of the 4-day feeding period, and since cell growth was not affected. The reduction of CTs was, at least in part, the result of transcriptional down-regulation of phenylpropanoid and flavonoid pathway genes, a process known to be integrated with sugar sensing [35]. In salicyl alcohol-fed cultures, multiple genes involved in sucrose transport and hydrolysis were up-regulated, including those encoding vacuolar sucrose transporter (*SUT4*), cytosolic sucrose synthases (*SuSy1, 2 *and *3*) and vacuolar invertase (*VIN2*). This suggests that altered compartmentalization of sucrose hydrolysis along with increased glycosylation may have altered phenylpropanoid flux in the fed cells.

Genes involved in the Krebs cycle and glycolysis were also up-regulated in salicyl alcohol-fed cultures, implicating a diversion of metabolites into the Krebs cycle through glycolysis. One of the important processes supported by the Krebs cycle is respiration. Increased respiration is associated with increased carbon loss as CO_2_. Whether this was linked to reduced CT synthesis seems unlikely due to the high availability of external sucrose. The Krebs cycle also utilizes acetyl-CoA, a precursor for malonyl-CoA. As malonyl-CoA contributes 40% of the carbon comprising CT, elevated Krebs cycle metabolism could reduce the amount of carbon allocated for CT synthesis. The Krebs cycle supplies energy as well as carbon for biosynthetic intermediates that are required for growth. If the gene up-regulation we observed was related to growth maintenance, it would mean that growth outcompeted, or had a higher priority over CT synthesis for cellular carbon in salicyl alcohol-fed cells. Whether salicyl alcohol feeding reduced cellular uptake of sucrose, thereby limiting cellular resources and CT accrual was not determined.

### Salicyl alcohol glycosylation and transport

Essentially all of the GT1 members found to be well-expressed in cell cultures were up-regulated by salicyl alcohol feeding. The transcript levels of *GT1-2 *and *GT1-246 *were especially high, and nearly doubled in salicyl alcohol-fed cells. The data suggest that they may mediate the glycosylation of salicyl alcohol into salicin. Alternatively, because many GTs exhibit broad substrate specificity, sometimes functioning in xenobiotics detoxification [36], up-regulation of well-expressed GT1 genes might merely reflect stimulation due to a possible stress of salicyl alcohol feeding. Up-regulation of several defense-related genes in the fed cells lends further credence to a general stress response. Unequivocal identification of GT1s that specifically regulate PG accrual will require a system that synthesizes aglycone PG precursors. Nevertheless, the work reported here offers glycosylation as an important determinant of the partitioning of carbon among pathways that begin with hydroxycinnamate and benzoate substrates. After glycosylation, glycosides are likely transported and sequestered into the vacuole for storage [26-28]. The up-regulation of putative tonoplast-localized MRP transporters, *MRP1 *and *MRP6*, suggests their possible involvement in the transport of salicins from cytoplasm into vacuole. Salicin transport may also be mediated by vacuolar localized SUTs [19], which could implicate the possible involvement of *Populus *SUT4 as well.

## Conclusions

We show that exogenous salicyl alcohol was readily glycosylated in aspen cell cultures at the expense of CTs. Transcriptome analysis revealed that genes involved in carbohydrate metabolism, glycolysis and the Krebs cycle were up-regulated by salicyl alcohol feeding, presumably to accommodate increased glycosylation while maintaining growth. Conversely, flavonoid pathway genes were down-regulated, consistent with reduced CT accrual. Given that glycosylation was the only PG biosynthetic step occurring in the fed cells, the metabolic tradeoff between CT and PG biosynthesis that we observed suggests that glycosylation of phenolic products contributes significantly to regulating the tradeoff between competing chemical defenses in intact plants. Future studies should target the salicyl alcohol-responsive genes, such as *GT1-2*, *GT1-246*, *MRP1*, *MRP6 *and *SUT4*, to determine their specific functions in PG synthesis and transport, and what role they play in the interaction of growth with CT and PG biosynthesis.

## Methods

### Cell cultures

Calli were induced from surface-sterilized leaves of greenhouse-grown *Populus tremuloides *genotype L-4 on semi-solid Woody Plant Medium [37] supplemented with 2.2 mg l^-1 ^of 2,4-dichlorophenoxy acetic acid and 3% sucrose. Suspension cultures were established with ~5 gm calli in 30 ml of liquid media in a 125 ml flask, and maintained in an orbital shaker at 120 rpm in the dark at 25°C. Cells were subcultured at 11-day intervals by inoculating 5 ml culture to 30 ml fresh medium. When indicated, replicate cultures were maintained in nephelo flasks for estimation of culture growth by the settled cell volume. Prior to measurement, approximately 10 ml of cell suspensions were allowed to settle for 25 min in the sidearm of the nephelo flask. The fraction of the suspension occupied by the cells was determined as the percent settled cell volume.

### Feeding experiments

Phenolic compounds with concentrations ranging from 0.2 to 5 mM were administered to the cultures at 5 DAS unless otherwise specified. Phenolic compounds were dissolved in DMSO for use in initial feeding trials, and feeding volumes ranged from 12 to 32 μl per flask. Control flasks received blank DMSO, and DMSO alone (with variations in feeding volumes) did not lead to accumulation of the various glucosides of interest. In subsequent feeding trials, salicyl alcohol was dissolved in water and feeding volumes ranged from 60 to 600 μl per flask for the dose-dependent experiments (control flasks received 300 μl of water). For the time-course (culture age) study and the microarray analysis, salicyl alcohol (5 mM final concentration) or water was administered at a fixed volume of 300 μl. Cells were harvested at regular intervals by low vacuum filtration, snap frozen in liquid nitrogen and stored at -80°C until use.

### PG and CT analysis

Freeze-dried samples (5 mg each) were extracted in 800 μl of cold methanol for 20 min in a cold ultrasonic bath and centrifuged at 15,000 *g *for 5 min. The methanol extracts (5 μl) were injected into an Eclipse XBD-C18 column (5 μm, 2.1 × 150 mm) and analyzed by HPLC-UV/MS (Hewlett-Packard 1100 Series, Agilent Technologies) at a flow rate of 0.2 ml/min using solvents A (10 mM formic acid, pH 3.4) and B (100% acetonitrile) according to the following gradient: 0 to 15 min, 0% to 70% B, 15 to 17 min, 70% to 100% B, 17 to 19 min, 100% to 0% B, and 19 to 30 min, 0% B. Glucosides (salicin, isosalicin, cinnamoyl-glucoside, *O*-coumaroyl-glucoside, salicyloyl-glucoside, benzyl alcohol-glucoside, benzoyl-glucoside, and helicin) were identified by UV absorbance and mass spectral data (Additional file 1). Concentrations of both salicin and isosalicin were estimated by a calibration curve developed using authentic salicin (Sigma). Total CTs were estimated as described [32] according to Porter et al., [38], using a standard curve developed based on purified aspen leaf CTs.

### Microarray analysis

RNA was extracted from frozen cells according to Chang et al. [39] and treated with Turbo DNase according to the manufacturer's instructions (Ambion). Aminoallyl-labeled cDNA synthesis, Cy-dye coupling, purification, EST microarray processing were carried out as described [32], except for hybridization which was performed in a humid hybridization oven (Boekel Scientific). An equal amount (50 pmole) of Cy3- and Cy5-labeled cDNA from control and salicyl alcohol-fed cells was mixed and used for each hybridization. The experiment consisted of three biological replicates (three controls randomly paired with three treatment groups) with a technical dye swap for each pair of samples, giving rise to a total of 6 hybridizations.

Hybridized slides were scanned with a Genepix 4000B scanner (Axon Instruments) and the florescence signal intensity was quantified using the GenePix Pro 5.1 software (Axon Instruments). Probes with signal intensities in both channels greater than two standard deviations from the background signal were flagged as present, and other irregular or low-quality spots were manually flagged. Data were normalized by theLOWLESS (locally weighted linear regression) algorithm implemented in the GeneSpring 7.3.1 software (Agilent). Probes (excluding spike controls) that passed the following quality control measures were subjected to statistical analysis: present in at least four replicates (4,132 spots) and with a coefficient of variation among replicates less than 35% (3,957 spots). A total of 1,157 ESTs were found to be differentially expressed based on *t-*test, with a Benjamini and Hochberg false discovery rate for multiple testing correction at *P *≤ 0.05. The list was further filtered to retain 444 probes with (1) a raw hybridization signal ≥ 100 in at least four of the six replicates (725), and (2) a fold-change cutoff of 1.3. The microarray data has been submitted to GenBank Gene Expression Omnibus repository under accession number GSE18360.

### Q-PCR expression analysis

cDNA was synthesized using DNA-free total RNA, anchored oligo(dT)20 primers and SuperScript II reverse transcriptase (Invitrogen). Relative transcript abundance was analyzed by Q-PCR using cDNA derived from 2.5 ng of total RNA, gene-specific primers and ABsolute QPCR SYBR Green Mix (ABgene) with ROX as an internal reference. Relative expression of the genes of interest was calculated by the ΔC_T _method as described [6] using ubiquitin-conjugating enzyme E2 and elongation factor 1-β as the housekeeping genes. Primer information is provided in Additional file 3, except phenylpropanoid gene primers as reported [6].

## List of abbreviations

4CL: 4-coumarate:CoA ligase; ANR: anthocyanidin reductase; ANS: anthocyanidin synthase; C4H: cinnamate 4-hydroxylase; CHI: chalcone isomerase; CIN: cell wall invertases; CT: condensed tannin; DAS: days after subculture; F3H: flavanone 3-hydroxylase; GT: glycosyltransferase; NIN: neutral invertases; MRP: multidrug resistance protein; PAL: phenylalanine ammonia-lyase; PG: phenolic glycoside; SuSy: sucrose synthase; SUT: sucrose transporter; TCA: tricarboxylic acid; VIN: vacuolar invertase.

## Authors' contributions

CJT, RSP and SAH designed the research, RSP and MPN performed the experiments, RSP, BAB, SAH and CJT wrote the manuscript. All authors read and approved the manuscript.

## Supplementary Material

Additional file 1 **HLPC-MS characteristics of various glucosides detected in the cell culture feeding experiments**. Precursors used and products detected in the feeding experiments. Click here for file

Additional file 2 **List of differentially expressed ESTs in salicyl alcohol-fed cells**. ESTs with an expression ratio greater than 1.3 in salicyl alcohol-fed cells relative to unfed cells. Click here for file

Additional file 3 **List of gene-specific primers used in QPCR analysis**. primer table. Click here for file
