# Supplementary material for: Glycosylation-mediated phenylpropanoid partitioning in Populus tremuloides cell cultures
Source: BMC Plant Biol. 2009 Dec 29;9:151. doi: 10.1186/1471-2229-9-151 (PMC2808312; doi:10.1186/1471-2229-9-151)
Supplement: Additional file 1 — HLPC-MS characteristics of various glucosides detected in the cell culture feeding experiments. Precursors used and products detected in the feeding experiments. [file 1471-2229-9-151-S1.DOC]

**Additional file 1: HPLC-MS characteristics of various glucosides detected in the cell culture feeding experiments.**

| **Precursor** | **Product formed** | ***m/z*** | **Retention time (min)** | **λ_max_ (nm)** |
| --- | --- | --- | --- | --- |
| Salicin | Salicin (formide adduct) | 331 | 10.3 | 268 |
|  | Isosalicin | 285 | 10.8 | 274 |
| Helicin | Salicin and Isosalicin | same as above | | |
| Salicyl alcohol | Salicin and Isosalicin | same as above | | |
| Salicylaldehyde | Salicin and Isosalicin | same as above | | |
| Salicylic acid | Salicylic acid glucoside | 299 | 14.8 | 282 |
| Benzyl alcohol | Benzyl alcohol-glucoside (formide adduct) | 315 | 11.9 | 285 |
| Benzylaldehyde | Benzyl alcohol-glucoside | same as above | | |
| Benzoic acid | Benzoyl-glucoside (formide adduct) | 329 | 12.04 | 234; 276 |
| Cinnamic acid | Cinnamoyl-glucoside (formide adduct) | 355 | 13.72 | 282 |
| *O*-Coumaric acid | *O*-coumaroyl-glucoside or  *O*-coumaric acid glucose ester | 325 | 12.45; 12.86 | 279 |
