# Supplementary material for: Glycosylation-mediated phenylpropanoid partitioning in Populus tremuloides cell cultures
Source: BMC Plant Biol. 2009 Dec 29;9:151. doi: 10.1186/1471-2229-9-151 (PMC2808312; doi:10.1186/1471-2229-9-151)
Supplement: Additional file 3 — List of gene-specific primers used in QPCR analysis. primer table. [file 1471-2229-9-151-S3.DOC]

Additional file 3. List of gene-specific primers used in QPCR analysis.

| **Gene** | **Primer sequence** |  | **Gene** | **Primer sequence** |
| --- | --- | --- | --- | --- |
| NIN2/5 | TGCTTTGGCYGAGAAGAGACTTMA |  | GT1-225 | AGACTGTTGGTGGAGGAATTGGGA |
|  | GGCCACTHGTGTTAAGYCCACAAA |  |  | ATTCCTYGATGAGCCCGTTCAAGT |
| NIN3/4 | AACAAGCACRYCTGTTCCAGACAT |  | GT1-200 | TGGTGCCCTGGAATGGTCWAGATA |
|  | TCTGWCCACGMTTTCTCCTTGGRT |  |  | CTGCGGATTCTTGCTTGCTTGTC |
| NIN8/12 | TTTATGGTTGCTGACTGCTGCRTG |  | GT1-292 | ACTGCAGGGAARCCMATGRTTACA |
|  | CCAGCATCATYTTTGCCACCAAGT |  |  | TCCACCTTCTTCRACAGCCTTCCT |
| NIN9/11 | GCTTCTCACMGCRGCATGCATMAA |  | GT1-298 | TGAAGATTGGTGTRAGGGTTGGGT |
|  | TCYTCGAGTGCCACCRTRCCCAAA |  |  | AGCCCTCTTCTTCCACTGCTTTGT |
| CIN1/2 | AGAAYTGCCATCWCATCYAGGGTT |  | GT1-246 | AGTGCTGAACCATCCATCGGTAGG |
|  | TTCATTGCGTGGATTCTCYCCC |  |  | CTGTGGTTCGCTCCCTAATCAACT |
| CIN3 | AGAAYTGCCATCWCATCYAGGGTT |  | GT1-258 | GCTCGCAAGTGGAAGAACCACAAA |
|  | GTACGGGACAATTTCATCATCCATTG |  |  | TCACGTGGCAGAGAGCACMTTATT |
| CIN4 | GCTATTCAWGAAGAAGCTCGCCTG |  | GT1-262 | CTGCCTGAAGGTTTCTTGGAACGA |
|  | AGACACTAAAGCAGACTGCAGAGG |  |  | ACCTTCTTCTCCACCTCATCAGCA |
| CIN5 | TTTCGTAGACATGGATCCTCGCCA |  | GT1-289 | TTGCTACATGGCCAATGCATGCTG |
|  | TGGCTTCCTTCGTTTCGTGGTAGA |  |  | CACACGATSTAACCAAGTGKAYGAGG |
| VIN1 | TTATCCGACGASGGCAATMTATGG |  | GT1-188 | TGMCTATGCTTTGTTGGCCKTCCT |
|  | GGTACAGATGGATGCAAATTAGGT |  |  | WTGACAARAGCACTTCCGTCACCA |
| VIN2 | TTATCCGACGASGGCAATMTATGG |  | GT1-253 | TTGGTGTCCACAAGAGGAAGTKCT |
|  | CGAAGGCABTGCTACTGTTKTTCA |  |  | TGAACCATTAGGGACAGTAGCCTC |
| VIN3 | AGGCCACACTCAAGATTTGGGA |  | GT1-270/274 | CCTATGCTTTGTTGGCCATTCGCT |
|  | TCTCAYGTGGTTGCCTCAAGGT |  |  | AAGTTCATGGATGATGAACCACCGGG |
| SUSY1 | GAACCTTGATCGTCTTGAGAGYCG |  | GT1-324 | CCCATGATTTGCTGGCCCTTCTTT |
|  | GGTTCTGTCTCCMAACYGAAACCA |  |  | GCAATTCACTGACCAGCTTCTCCA |
| SUSY2 | CAACCTYGATCAYCGTGAGAGCCG |  | GT1-109 | CTGTGCCATTCTGCTGTTGGAGGT |
|  | ACCATTATTCTGGACCCGGAACCC |  |  | AGCTCTCTTCCGCATTGCTTTCAC |
| SUSY3 | TATCTGATGCTGGGCTKCAACGGA |  | GT1-186 | GGAGMGAAGGGCGAAGAGATGARA |
|  | TGCCRGTCMTCGATTGACAAAGGT |  |  | ACATCRCAGTCCARAGAAGRAAGG |
| SUSY4 | CAATCAAGGTGGCCCAGCAGAAAT |  | GT1-2 | AATGCCAAGYTGATTGMAGACGTG |
|  | GTAGATGCGTTGGAGACCAGTTGC |  |  | ACCCRTCCATCATAACAACCAAGC |
| SUSY5 | CAATCAAGGTGGCCCAGCAGAAAT |  | GT1-221 | AACACTTGAGGCTTTGAGCTTGGG |
|  | ATAGATGCGTTGAAGACCAGCTGC |  |  | AGCTTCCACCTTCATCCATAGCCA |
| SUSY6 | TGGATCCCGGACACTGGAATAAGT |  | GT1-315 | CCTAAGGCAGTGGAAATGAAGSAG |
|  | TCTGAGRCTGGTGTTTGASCTTCT |  |  | CCAGTTCADCSACTTYCTCRHTGG |
| SUT1/2 | TGGTKTCTGTAGCRRSTGGACCTT |  | GT1-293 | TGGGTGGAATTCTGTCCTTGAAGC |
|  | ACCAGTCACCAGTCTTGGAAGGAA |  |  | CCCTTGACCTTGCTAGCTTTCCTT |
| SUT3 | TGGTKTCTGTAGCRRSTGGACCTT |  | MRP1 | ATATCCAGAGATGGGTGGTGGTCA |
|  | GGAATGCAKCAGTGACAGYCMTTT |  |  | CAGGTACTTGCAGTAAGTTGCTACC |
| SUT4 | ATCCTTGGGACTTGGACAAGGGTT |  | MRP2 | AGGAAGCGCATTCTCTAGGATGGT |
|  | TGATCGAGGAATACYCAAGATGGC |  |  | AGCAAGATGCATAAGACACGACA |
| SUT5 | ATACCAGCSTTYGTTCTGGCWTCT |  | MRP3 | AGGAAGCGCATTCTCTAGGATGGT |
|  | TAGCATGCTCCTGTCCTTGACAATYA |  |  | GAGACTAACAACCAGGGCSAGTT |
| SUT6 | ATACCAGCSTTYGTTCTGGCWTCT |  | MRP4 | AGTTGATTCRCAAACTGAYGCTG |
|  | TCCTYGACRATTACATGTTGGCTC |  |  | AAACAGYGATGGCCTCTCAAGCAA |
| EF1B | AAGAGGACAAGAAGGCAGCA |  | MRP5 | GTGCTTGATGAAGCRACRGCATCT |
|  | CTAACCGCCTTCTCCAACAC |  |  | ATCCRGTAYGCYACTTGACCTTGA |
| UBCc | CTGAAGAAGGAGATGACARCMCCA |  | MRP6 | CACAGTAGCTCACMGGATACCRAC |
|  | GCATCCCTTCAACACAGTTTCAMG |  |  | CTTCAATGYGATTCTGCWRCRTGT |
